# Clopidogrel-Induced Hypersensitivity Syndrome Presenting as a Progressive Rash: A Case Report

**DOI:** 10.7759/cureus.112611

**Published:** 2026-07-13

**Authors:** Saviz Saghari, Marie Geha, Mason Arbabi, Hamayon Babary

**Affiliations:** 1 Internal Medicine, West Anaheim Medical Center, Anaheim, USA; 2 Internal Medicine, University of Arizona College of Medicine-Tucson, Tucson, USA; 3 Rheumatology, Facey Medical Group, Mission Hills, USA

**Keywords:** antiplatelet therapy, clopidogrel hypersensitivity, corticosteroids, erythematous rashes, hypersensitivity reaction

## Abstract

Clopidogrel is a commonly prescribed antiplatelet agent used to prevent thrombotic events, particularly in patients with cardiovascular disease. While effective, hypersensitivity reactions, though rare, can complicate its use. We present the case of a 70-year-old male with a history of coronary artery disease who developed a progressive bilateral lower extremity rash following long-term clopidogrel use. Laboratory findings revealed elevated inflammatory markers, and a skin biopsy showed superficial perivascular lymphocytic infiltrate with rare eosinophils. The patient was diagnosed with clopidogrel-induced hypersensitivity, leading to discontinuation of the drug and initiation of ticagrelor. A short course of systemic corticosteroids and antihistamines resulted in symptom resolution within two weeks.

## Introduction

Clopidogrel is a widely used antiplatelet agent belonging to the thienopyridine class, primarily prescribed to reduce the risk of thrombotic events in patients with cardiovascular diseases, particularly those undergoing percutaneous coronary interventions (PCI) or diagnosed with acute coronary syndromes [1]. It functions by irreversibly inhibiting the P2Y12 receptor on platelets, preventing platelet aggregation and reducing the likelihood of arterial thrombosis [2]. The efficacy of clopidogrel in preventing cardiovascular events has been extensively demonstrated in clinical trials, including the Clopidogrel versus Aspirin in Patients at Risk of Ischemic Events (CAPRIE) and Clopidogrel in Unstable Angina to Prevent Recurrent Events (CURE) trials, leading to its widespread use in secondary prevention strategies [3]. However, despite its benefits, clopidogrel has been associated with several adverse effects, the most common being an increased risk of bleeding. Other less frequent but clinically significant complications include gastrointestinal disturbances, hematologic abnormalities, and hypersensitivity reactions [4].

Hypersensitivity reactions to clopidogrel, though uncommon, have been increasingly recognized in clinical practice, with reported incidence rates ranging from 0.5% to 1% of treated patients [5]. These reactions typically manifest as cutaneous symptoms such as maculopapular rashes, urticaria, or angioedema. In rare instances, more severe systemic hypersensitivity responses, including drug reaction with eosinophilia and systemic symptoms (DRESS) syndrome and anaphylaxis, have been reported [6]. Although most hypersensitivity reactions are described shortly after treatment initiation, atypical or delayed presentations may create diagnostic uncertainty, particularly when symptoms occur after prolonged drug exposure or in the setting of other inflammatory triggers. We present the case of a 70-year-old man with long-term clopidogrel exposure who developed a progressive bilateral lower-extremity purpuric rash, highlighting the diagnostic challenges of attributing delayed cutaneous reactions to a long-standing medication and the management considerations required to maintain antiplatelet protection.

## Case presentation

A 70-year-old male with a past medical history of coronary artery disease (CAD) and myocardial infarction (MI) status post percutaneous coronary intervention (PCI) with four stents, hypertension (HTN), hyperlipidemia (HLD), and an unspecified liver disease presented with a progressive bilateral lower extremity rash. The patient had been on clopidogrel (Plavix) for 14 years. No recent interruption or re-initiation of clopidogrel was documented in the available record. No recent medication changes were identified; however, information on a possible manufacturer or formulation change was unavailable.

Three days prior to presentation, he noted the onset of bilateral raised, erythematous rashes on the dorsum of his feet, which rapidly extended to his shins (Figure 1). He reported associated burning pain and pruritus, with increasing difficulty in ambulation over the past two days. The patient denied alcohol consumption, smoking, or illicit drug use.

**Figure 1:**
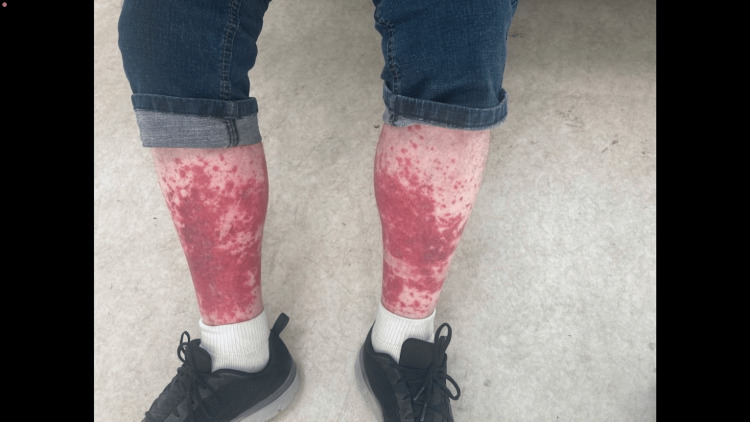
Bilateral lower-extremity purpuric macules and irregular patches, symmetrically distributed over the feet and legs, associated with pruritus and burning pain. The purpuric morphology prompted consideration of small-vessel vasculitis in the differential diagnosis.

Apart from the rash, the patient reported a dry cough but denied other systemic complaints. He recalled experiencing a fever two weeks prior, coinciding with an upper respiratory infection (URI), but denied fever at presentation. He denied arthralgia, abdominal pain, hematuria, oral ulcers, mucosal lesions, dyspnea, or other systemic symptoms suggestive of vasculitic or multisystem involvement.

On arrival, his vital signs were as follows: blood pressure 147/67 mmHg (left arm, sitting), heart rate 79 bpm, temperature 36.7°C (oral), respiratory rate 16 breaths/min, and peripheral oxygen saturation (SpO2) 97%. His height was 1.676 m, weight 78 kg, with a Body Mass Index (BMI) of 27.76 kg/m².

Laboratory investigations revealed a normal complete blood count (CBC) and comprehensive metabolic panel (CMP). Platelet count and renal function were within normal limits. Serologies for hepatitis A, B, and C, as well as HIV, were negative. Influenza A and B antigen tests were also negative. However, the C-reactive protein (CRP) was elevated at 22.2 mg/L. The complement C4 level was significantly reduced (Table 1). Urinalysis showed no hematuria or proteinuria, and chest radiography showed no acute cardiopulmonary abnormality.

**Table 1:** Laboratory results. CRP: C-reactive protein; DNA: deoxyribonucleic acid; RNP: ribonucleoprotein; SS-A: anti-Sjögren's-syndrome-related antigen A; SS-B: anti-Sjögren's-syndrome-related antigen B; INR: international normalized ratio; AI: antibody index; IU: international index.

Test	Result	Normal range
CRP	22.2 mg/L	<10 mg/L
Complement C4	<2 mg/dL	12–38 mg/dL
Complement C3	130 mg/dL	82-167 mg/dL
Anti-DNA	2 IU/mL	0-9 IU/mL
Antinuclear antibodies	Negative	<1:80
Antiparietal cell antibody	7.1 Units	0-20 Units
RNP antibodies	<0.2	0-0.9 AI
Anti-Smith antibodies	<0.2	0-0.9 AI
Actin	3 Units	0-19 Units
Mitochondrial (M2) antibody	<20 Units	0-20 Units
Antiscleroderma antibodies	<0.2 AI	0-0.9 AI
Sjogren's anti-SS-A	<0.2 AI	0-0.9 AI
Sjogren's anti-SS-B	<0.2 AI	0-0.9 AI
Antichromatin antibodies	<0.2 AI	0-0.9 AI
Anti-Jo-1	<0.2 AI	0-0.9 AI
Anti-centromere-B antibodies	<0.2 AI	0-0.9 AI
Anti-striation antibodies	Negative	<1:100
INR	1.01	0.9-1.1
Erythrocyte sedimentation rate (ESR)	10 mm/h	0-20 mm/h
Anti-thyroid peroxidase (TPO) antibodies	<9 IU/mL	0-34 IU/mL

The patient was administered 125 mg of intravenous (IV) methylprednisolone and subsequently admitted for further evaluation and management.

Skin biopsy revealed benign skin with superficial perivascular lymphocytic infiltrate with rare eosinophils (Figure 2). No definitive leukocytoclastic vasculitis was reported in the available histopathologic description.

**Figure 2:**
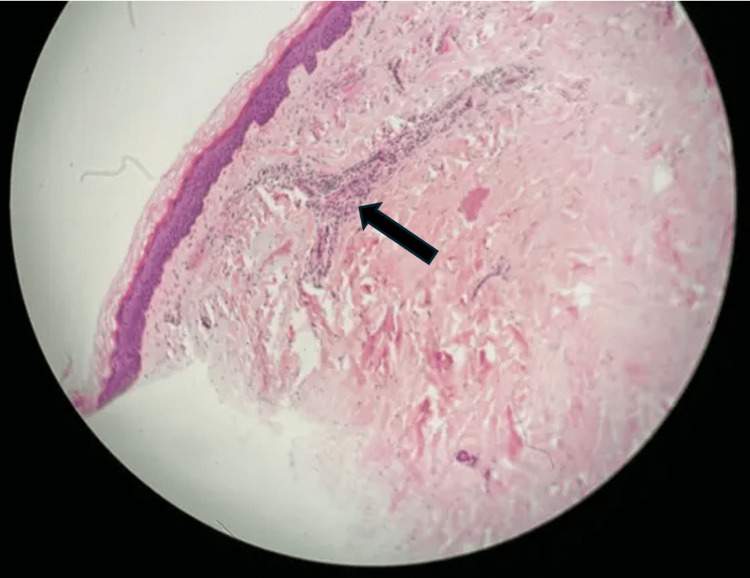
Skin biopsy showing superficial perivascular lymphocytic infiltrate with rare eosinophils, a nonspecific pattern that may be seen in drug-related cutaneous hypersensitivity reactions. No definitive leukocytoclastic vasculitis was reported in the available histopathologic description.

Given the suspicion of a clopidogrel-induced hypersensitivity reaction, the medication was discontinued. The patient was initiated on oral antihistamines and a short course of systemic corticosteroids to manage the inflammatory response. Alternative antiplatelet therapy was initiated with ticagrelor 90 mg orally twice daily to maintain P2Y12 inhibition and reduce thrombotic risk. Supportive care included topical emollients and corticosteroid creams for symptom relief. Over the following days, the patient demonstrated significant improvement in rash and pruritus, with resolution of burning pain. Inflammatory markers showed a downward trend, and no new symptoms were reported.

The patient was discharged with a plan for continued outpatient follow-up, emphasizing medication adherence and monitoring for recurrent reactions. At a two-week follow-up visit, his skin lesions had resolved completely, and he remained stable without further complications.

## Discussion

Clopidogrel is a widely prescribed antiplatelet agent used to prevent thrombotic events in patients with cardiovascular disease. Although its efficacy is well established, clopidogrel may be associated with adverse effects, including hypersensitivity reactions. These reactions are relatively uncommon, with reported incidence rates of approximately 0.5%-1% among treated patients [1]. Clinical manifestations range from mild cutaneous eruptions, such as maculopapular rash, urticaria, and pruritus, to less frequent systemic reactions [2].

The pathophysiology of clopidogrel-associated hypersensitivity is not fully understood but is generally considered to involve a delayed T-cell-mediated immune response, corresponding to a type IV hypersensitivity mechanism. Histopathologic findings may include superficial perivascular lymphocytic infiltrates, sometimes with eosinophils, supporting a drug-related hypersensitivity pattern [3]. However, these biopsy findings are not specific and should be interpreted in the context of the clinical presentation, medication history, and exclusion of alternative causes. Mast-cell activation and inflammatory mediator release have also been implicated in drug-related hypersensitivity phenomena, including Kounis syndrome, although this patient did not present with features suggestive of acute coronary vasospasm or allergic acute coronary syndrome [4].

Diagnosis of clopidogrel-associated hypersensitivity relies on careful assessment of the temporal relationship between drug exposure and symptom onset, clinical morphology, histopathologic findings, response to drug withdrawal, and exclusion of mimicking conditions. Patch testing may support the diagnosis of delayed hypersensitivity in selected cases, but it is not routinely required and was not performed in the present case [5]. In patients with purpuric lower-extremity lesions, the differential diagnosis should be broadened beyond simple drug eruption to include leukocytoclastic vasculitis, infection-associated small-vessel vasculitis, cryoglobulinemic vasculitis, connective tissue disease, thrombocytopenic purpura, and coagulopathy-related purpura.

In the present case, a 70-year-old man with coronary artery disease and long-term clopidogrel exposure developed a progressive bilateral purpuric lower-extremity rash associated with burning pain and pruritus. Laboratory findings demonstrated elevated C-reactive protein and markedly reduced complement C4, while infectious testing for hepatitis A, B, and C, HIV, and influenza A/B was negative. The skin biopsy showed superficial perivascular lymphocytic infiltrate with rare eosinophils, a pattern compatible with a drug-related cutaneous hypersensitivity reaction but not pathognomonic. Importantly, definitive histopathologic features of leukocytoclastic vasculitis, such as vessel wall necrosis, leukocytoclasia, or fibrinoid change, were not reported in the available biopsy description.

The markedly reduced C4 level with preserved C3 was an atypical finding for an uncomplicated exanthematous drug eruption and broadened the differential diagnosis to include immune-complex-mediated or complement-mediated processes. However, autoimmune serologies were negative, viral hepatitis and HIV testing were negative, and the available clinical evaluation did not identify clear renal, pulmonary, mucosal, or multisystem involvement. Therefore, the low C4 was interpreted with caution as a nonspecific marker of immune activation rather than definitive evidence of a specific alternative diagnosis.

A major diagnostic challenge in this case was the unusually long latency, as the patient had been taking clopidogrel for approximately 14 years before symptom onset. Most clopidogrel hypersensitivity reactions are reported soon after treatment initiation. Therefore, causality cannot be inferred from chronology alone. Possible explanations include delayed immune sensitization, an unrecognized change in drug formulation or manufacturer, cumulative immune threshold effects, or an inflammatory “second hit” related to the recent upper respiratory infection. The preceding upper respiratory infection may also represent an alternative or contributing trigger for the eruption and was therefore considered in the diagnostic reasoning.

To strengthen causality assessment, the Naranjo adverse drug reaction probability scale was applied. The patient received points for previous reports of clopidogrel hypersensitivity, occurrence of the event after drug exposure, clinical improvement after discontinuation and treatment, and objective supportive evidence from skin biopsy. Points were limited by the absence of rechallenge, the atypically prolonged latency, and the possible contribution of a recent infection. Overall, the assessment supported a probable rather than definite clopidogrel-associated adverse reaction.

Management of clopidogrel hypersensitivity depends on the severity of the reaction and the patient’s thrombotic risk. In selected mild cases, continuation of clopidogrel with a short course of corticosteroids and antihistamines has been reported [6]. However, in moderate or progressive reactions, discontinuation of clopidogrel may be necessary. Switching to an alternative P2Y12 inhibitor, such as ticagrelor or prasugrel, can be considered, as these agents belong to different chemical classes and may have lower cross-reactivity [7]. In patients who require clopidogrel because of high thrombotic risk or lack of suitable alternatives, desensitization protocols may be performed under close medical supervision [8].

In this case, clopidogrel was discontinued, and ticagrelor was initiated to maintain antiplatelet protection. The patient also received systemic corticosteroids, oral antihistamines, topical corticosteroids, and supportive skin care. Symptoms improved over the following days, with complete resolution at the two-week follow-up. However, improvement cannot be attributed solely to clopidogrel withdrawal because systemic corticosteroids and antihistamines likely contributed to the rapid clinical response. The favorable outcome supports an inflammatory or hypersensitivity-mediated process but does not prove clopidogrel as the sole cause.

The prognosis of clopidogrel-associated hypersensitivity is generally favorable when recognized early and managed appropriately. Prior reports have described successful symptom control with corticosteroids without interrupting clopidogrel in selected cases, while others have demonstrated the feasibility of desensitization protocols when continued clopidogrel therapy is essential [1,8,9]. Close follow-up remains important to monitor for recurrence, ensure tolerance of alternative antiplatelet therapy, and reassess for systemic features if symptoms recur.

## Conclusions

Clopidogrel-associated cutaneous hypersensitivity is uncommon but remains a clinically important adverse reaction requiring early recognition and appropriate management. This case describes a probable clopidogrel-associated delayed cutaneous hypersensitivity reaction presenting as a progressive bilateral purpuric lower-extremity rash after prolonged clopidogrel exposure. The unusually long latency, isolated low C4 level, recent upper respiratory infection, and nonspecific histopathologic findings made causality assessment challenging and required consideration of infectious, autoimmune, hematologic, and vasculitic mimics. Discontinuation of clopidogrel, substitution with ticagrelor, and short-term corticosteroid and antihistamine therapy were followed by complete clinical resolution. Clinicians should consider both newly introduced and long-standing medications when evaluating unexplained purpuric eruptions, while applying cautious causality assessment before attributing symptoms to a single drug.
